# The Gut-Brain Axis in Alzheimer’s Disease and Omega-3. A Critical Overview of Clinical Trials

**DOI:** 10.3390/nu10091267

**Published:** 2018-09-08

**Authors:** Francesca La Rosa, Mario Clerici, Daniela Ratto, Alessandra Occhinegro, Anna Licito, Marcello Romeo, Carmine Di Iorio, Paola Rossi

**Affiliations:** 1 IRCCS Fondazione Don Carlo Gnocchi, 20100 Milan, Italy; larosa.francy@gmail.com (F.L.R.); mario.clerici@unimi.it (M.C.); 2 Department of Physiopathology and Transplants, University of Milano, 20100 Milan, Italy; 3 Department of Biology and Biotechnology, University of Pavia, 27100 Pavia, Italy; daniela.ratto@gmail.com (D.R.); alessandraocchinegro@hotmail.it (A.O.); drmarcelloromeo@gmail.com (M.R.); carminediiorio01@universitadipavia.it (C.D.I.); 4 Istituto per lo Studio e la Cura del Diabete [ISCD], Casagiove, 81022 Caserta, Italy; arianna.licito@hotmail.it

**Keywords:** Alzheimer’s disease, neuroinflammation, microbiota, neurodegeneration, cognitive impairment, omega-3

## Abstract

Despite intensive study, neurodegenerative diseases remain insufficiently understood, precluding rational design of therapeutic interventions that can reverse or even arrest the progressive loss of neurological function. In the last decade, several theories investigating the causes of neurodegenerative diseases have been formulated and a condition or risk factor that can contribute is described by the gut-brain axis hypothesis: stress, unbalanced diet, and drugs impact altering microbiota composition which contributes to dysbiosis. An altered gut microbiota may lead to a dysbiotic condition and to a subsequent increase in intestinal permeability, causing the so-called leaky-gut syndrome. Herein, in this review we report recent findings in clinical trials on the risk factor of the gut-brain axis in Alzheimer’s disease and on the effect of omega-3 supplementation, in shifting gut microbiota balance towards an eubiosis status. Despite this promising effect, evidences reported in selected randomized clinical trials on the effect of omega-3 fatty acid on cognitive decline in Alzheimer’s disease are few. Only Mild Cognitive Impairment, a prodromal state that could precede the progress to Alzheimer’s disease could be affected by omega-3 FA supplementation. We report some of the critical issues which emerged from these studies. Randomized controlled trials in well-selected AD patients considering the critical points underlined in this review are warranted.

## 1. Introduction

The gut microbiota is defined as the microbial community found within the gut. This microbial ecosystem is an essential endocrine “organ” involved in critical functions such as influencing metabolism and the absorption of food, providing trophic and protective functions, instructing innate immunity, and acting as a dynamic filter of environmental exposure in the host [1,2,3,4]. 

In healthy people, a study of 16S rRNA genes from human faecal microbial communities has shown that the three million bacteria of the gut microbiota belong to two large phyla, the *Bacteroidetes* and the *Firmicutes*, but this includes several hundred species-level phylotypes [1,5]. 

The process of human gut colonization is initiated in utero by different microbial communities present in the amniotic fluid and placenta [6] and is stably maintained over the lifespan of an individual: as an example, the *Bacteroidetes*/*Firmicutes* phylum ratio remains remarkably stable over time [5]. The change of the relative abundance of each species in the intestinal microbial community can influence immune performance, physiological homeostasis, and healthy ageing [7,8,9].

In the past, microorganisms have not been considered as particularly relevant to brain development and function, nor in the pathophysiology of chronic brain diseases, such as Alzheimer’s disease (AD), Parkinson’s disease (PD), multiple sclerosis (MS) or mood disorders. The characterization of the healthy human microbiome and, in particular, of the gut microbiome (Human Microbiome Project Consortium, 2012) revealed that the gut is involved in regulating brain function and in shifting the balance between protective and pathogenic immune responses [10,11]. 

Despite evidences demonstrating tight interactions between gut-associated immune, enteric, endocrine, and nervous systems [12], psychiatric and neurological communities have long ignored gut–brain interactions as a possible target to treat brain diseases. 

In the first part of this review, we discuss several lines of evidence that highlight the role of the gut microbiota in the development of neurodegenerative diseases, and in particular AD, focusing on intestinal wellbeing as a possible therapeutic approach. About that, two recent reviews describe how microbiota influences brain function, cognitive decline and behaviour via gut microbiota-brain axis in dementia and AD [13,14]. Although different neurophatological hallmarks of AD, including amyloid plaques, genetic and environment identified, this research proposed a new context to investigate the risk factors to AD development: inflammation and gut–pathogens colonization. 

In the second part, we first critically overview the effect of omega-3 fatty acid (FA) supplementation on microbiota composition and then in AD. Recent works underline that both diet [15] and/or prebiotics supplementation [16,17] act in a positive changing microbiota composition and increase anti-inflammatory immune responses. We report different evidences that demonstrate the effects docosahexaenoic acid (DHA) and eicosapentaenoic acid (EPA) supplementation in increasing short chain fatty acids production (SCFA) shifting gut microbiota balance towards eubiosis condition. Despite this promising omega-3 effect, in AD the only evidence of healthy effect of omega-3 FA supplementation is a delay in the cognitive decline in Mild Cognitive Impairment (MCI) [18], also reviewed by Fiala et al. [19]. Indeed, MCI is a prodromal clinical state that could precede the progress to Alzheimer’s disease, and, in particular, elderly patients with MCI are at high risk of developing dementia and AD [20]. 

In conclusion, current literature offers several ideas to speculate about an important involvement of gut-microorganisms in neurodegenerative diseases and in particular in AD. This critical overview of clinical trials suggests that randomized placebo-controlled double-blinded clinical trials will be necessary to clarify the effect of omega-3 FA supplementation in well-selected AD patients.

## 2. Gut Microbiota–Brain Axis in Alzheimer’s Disease

### 2.1. The Gut Ecosystem: Profile and Immunity

In 2002, Hooper et al. described the importance of the unknown world of the bacterial ecosystem: 1013–1014 inhabit our bodies and the great majority of these microorganisms are hidden in the gastrointestinal tract [21]. Initially, little was known about this heterogeneous bacterial population; with more than 1000 bacterial species, six phyla are highly expressed: *Firmicutes* and *Bacteroidetes* make up 90%, while *Actinobacteria*, *Proteobacteria*, *Fusobacteri*a and *Verrucomicrobia* represent the remaining 10%. In addition, Rampelli et al. described the genetic profile of such complex communities and their relationship with viruses by means of metagenomic analysis [22]. 

The human microbiota plays a crucial role in the structural integrity and metabolic functions of the colonic mucosa through the production of short chain fatty acids (SCFAs). Acetate, propionate, and butyrate are the most abundant SCFAs present in the gut lumen, reaching millimolar concentration levels in the colon. 

Butyrate is synthesized in the colon via the fermentation of otherwise non-digestible fibers introduced with the diet. A condensation process of two acetyl-CoA molecules is required to produce butyryl-CoA and then butyrate [23,24]. This process also leads to the formation of intermediate molecules such as lactate, succinate or formate. Such intermediate molecules, as well as butyrate, are absorbed from the gut lumen and used by bacteria to survive and proliferate [25,26,27]. Butyrate plays a pivotal role in maintaining human intestinal health by being the major energy source for the colonic mucosa and is fundamental in regulating gene expression, differentiation, inflammation, apoptosis in host cells and cross-feeding interactions with *Bifidobacteria* [28,29,30,31]. 

In a recent review, Bourassa discussed the role of bacteria butyrate producers suggesting that a high-fiber diet may improve brain health because it increases *Firmicutes* bacteria; butyrate may exert a neuroprotective effect as a histone deacetylase inhibitor, but also as a ligand for G-protein–coupled receptors and as a stimulus for mitochondrial activity [27].

**Figure 1:**
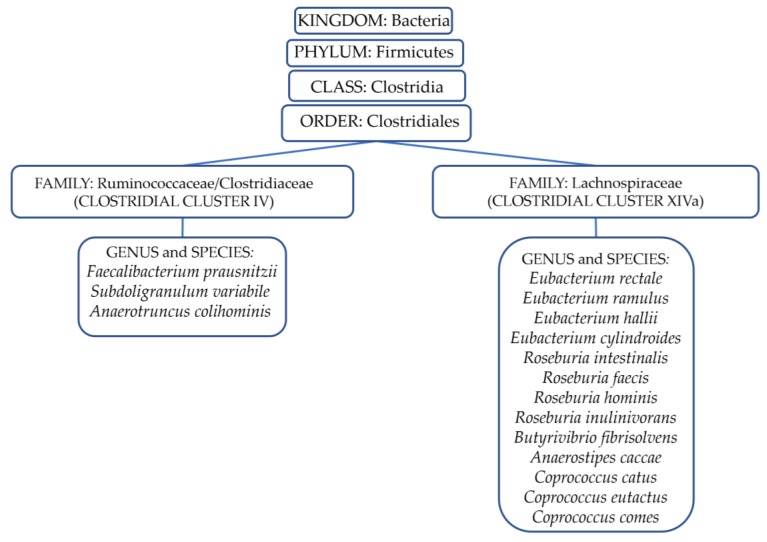
Taxonomic rank associated with dominant bacteria butyrate producers present in the human gut microbiota. All bacteria belong to the *Firmicutes* phylum. It is possible to divide these into two clusters by 16 rRNA analyses, each defined as a “clostridial cluster”. The first cluster is composed of *Rumnococcaceae* and *Clostridiaceae* bacteria, and the latter is composed of *Lachnospiraceae* bacteria. The production of butyrate preserves gut barrier functions, as well as exerts immunomodulatory and anti-inflammatory effects [29].

In Figure 1, we list the major bacterial genera associated with butyrate production. 

Intestinal microbiota colonization is essential in balancing immune responses in a homeostatic manner: studies conducted on germ-free mice suggest that the absence of an intestinal microbiota leads to impaired immune function (for a review see Round and Mazmanian, 2009, [32]) and that microbiota influences the specific phenotypes and physiological functions of T and B cells in the gut mucosa layer (for a review see Honda and Littman, 2016, [33]). These cells play a pivotal role in immune homeostasis by defending against foreign antigens and by maintaining the integrity of the gut mucosal barrier. A balanced gut-microbiota stimulates resident macrophages to release large amounts of interleukin (IL)-10 and transforming growth factor (TGF)-beta [34], thus promoting the induction of regulatory T cells (Tregs) and preventing an increase in the number of proinflammatory T helper 17 (Th17) cells in the gut [35]. In fact, a peripheral tolerance is maintained by the correct balance between gut-bacteria population and responses by the host.

An altered microbiota, including a reduction in health-promoting bacteria such as *Lactobacilli* and *Bifidobacteria*, may lead to a dysbiotic condition generated by Gram-negative-bacteria producing immunogenic endotoxins such as lipopolysaccharide (LPS) which increase intestinal permeability [33,36,37]. This condition changes the microbiota taxa profile and switches Treg to T-inflammatory cells (i.e., Th17) triggering a toll-like receptor (TLRs) mediated inflammatory response [38]. Very recently, the intestinal microbiota has emerged as a potential triggering factor in central nervous system–specific auto-immunity disease, as demonstrated in an experimental animal model for MS [39,40,41]. Gut microbiota from patients with MS enabled a spontaneous experimental autoimmune encephalomyelitis in germ-free transgenic mice, devoid of resident microbes, demonstrating that the MS-derived microbiota has factors that may precipitate an MS-like autoimmune disease in a transgenic mouse model [39]. Brain autoimmunity critically depends on the presence of an intact gut flora. Some members of the intestinal microbiota may ignite innate immune responses with the activation of dormant brain-specific T cells within gut-associated lymphatic tissues. Upon activation, such lymphocytes travel to the central nervous system (CNS) and trigger a cascade of events that culminate in the formation of brain-specific autoantibodies. Brain-specific autoantibodies together with T-cell activation produce demyelinating lesions, similar to active multiple sclerosis (MS) plaques [41]. 

As demonstrated in a recent pilot study conducted in our laboratory, dietary components, and in particular fruit, vegetables and fibers, allow the longtime maintenance of gut–microbiota homeostasis. It was observed that patients with MS who followed a controlled diet showed a significant decrease of Th1 and Th17 cells compared to those that followed a Western diet. This evidence correlated with an increase in the gut microbiota population of *Lachnospiraceae* family (phylum *Firmicutes*, see Figure 1), which are butyrate producers [15].

Therefore, these results link the gut microbiota to a variety of brain disorders. Studies of the mechanisms elucidating this transition could have important consequences both for developing new therapeutic strategies for treatment and the diagnosis of human neurodegenerative diseases [33]. It is still unclear whether a dysbiotic microbial profile exists a priori or whether changes in the composition of microbiota occur after the onset of disease [42]. 

### 2.2. The Gut Microbiota and Neuroinflammation

Increasing clinical findings [13,14] reveal that the pathogenesis of many neurodegenerative diseases may depend on the gut microbiota and that the resident commensal microbiota modulates CNS autoimmunity [43,44], beyond neuroinflammation [45,46].

A common trait between theories investigating the causes of neurodegenerative diseases is the presence of neuroinflammation [47,48] that has been associated with activation of microglia and peripheral monocytes that cross the BBB. These cells produce inflammatory cytokines and several neurotoxic molecules, such as TNF-α and IL-1β [49,50,51,52,53,54] in an attempt to counteract the formation and/or extend misfolding of neuronal proteins and the formation of insoluble fibrillary aggregates [55,56,57]. 

AD is associated with impaired cognition and cerebral accumulation of amyloid-β peptides (Aβ). Indeed, recent studies published, respectively, by Italian and American researchers described interactions between brain protein misfolding and microbiota [58,59]. 

Larsen et al. [60,61] and Jordal et al. [62] have described an abundance of functional bacterial amyloids; amyloid is used by bacteria such as *Proteobacteria*, *Bacteroidetes*, *Chloroflexi*, *Actinobacteria* and *Firmicutes* as structural and adhesive material, toxin, and protection against host innate immunological defenses [63]. The human innate immune system recognizes bacterial amyloid proteins by using several pathways involving TLR 1/2, Nod-like receptor-3 protein (NLRP3), nuclear factor kappa-B (NF-kb), CD14, and inducible nitric oxide synthase [57,64,65]. Other misfolded proteins produced by bacteria could predispose to tissue damage and to the production of proinflammatory cytokines associated with the development of dementia [66]. 

Other Authors [67,68] have shown that in a mouse model of Huntington’s disease treatment with butyrate-like analogues such as phenylbutyrate, histone deacetylase inhibitor or sodium butyrate used to modulate transcription prevents neuronal death and lengthens the life of mice in a dose-dependent manner.

As described above, the gut microbiota could influence the integrity of the blood-brain barrier (BBB). For example, this can be observed in a microbiota dysbiotic condition that induces increased permeability of the gut (the so-called “leaky gut”) in both foetal and adult mouse brains. Braniste et al. [69] conducted a study in adult and embryonic animals showing the relationship between the lack of a normal gut flora in germ-free mice and increased BBB permeability. This study highlighted the importance of gut microbiota integrity on the CNS, especially in the progression of neurodegenerative diseases. Studies by Diaz et al. in mice have shown that the normal gut microbiota modulates brain development and subsequent adult behaviour, investigated as motor control and anxiety behaviours, suggesting that the microbial colonization process triggers cellular pathways that affect specific neuronal circuits [11]. Other authors have shown that the disruption or absence of the microbiota in mice impaired the function of the BBB and altered cortical myelination and hippocampal neurogenesis, decreased cognitive function and memory formation, and decreased social behaviour [70].

According to Alkasir et al. [13] that proposed a schematic representation of the gut-microbiota and neuroinflammation axis in AD, we can speculate that dysfunction of the gut epithelial barrier results in peripheral inflammation. This condition is linked with dementia onset because the production of inflammatory cytokines, like IL-1β, reduces Aβ phagocytosis, induces NLRP3-inflammasome activation with consequent release of NLRP3-related cytokines such as caspase-1 and IL-18 [71], thus increasing amyloid-beta deposits. 

Therefore, modulation of innate immune responses through changes in microbiota composition may exert healthy effects especially on cognitive decline in MCI patients, possibly slowing down or avoiding the progression to AD.

## 3. Omega-3 Fatty Acids and Gut-Brain Axis in Alzheimer’s Disease

### 3.1. Omega-3 Fatty Acids and Microbiota 

Omega-3 FAs are a class of polyunsaturated fatty acids derived from the double bond in third position, starting the count from the terminal carbon of the chain. The three most important physiological omega-3 FAs are described: EPA, alpha-linolenic acid (ALA), and DHA. FAs are precursors for neuronal components membranes, fluidity of cell membranes, signalling, neurotransmission and modulation of enzymatic activities [72,73].

Recent studies addressing the correlation between omega-3 FAs and the human gut-microbiota [74,75,76,77,78] provided new insight into the microbiota composition, evidencing how this depends from several environmental factors, such as diet, exposure to microorganisms and antibiotic or pharmacologic therapies (Table 1). However, in spite of this, it is still a highly debated issue. Below we reported systematic reviews that are currently shared by the scientific community regarding the impact of omega-3 on microbiota. 

**Figure 2:**
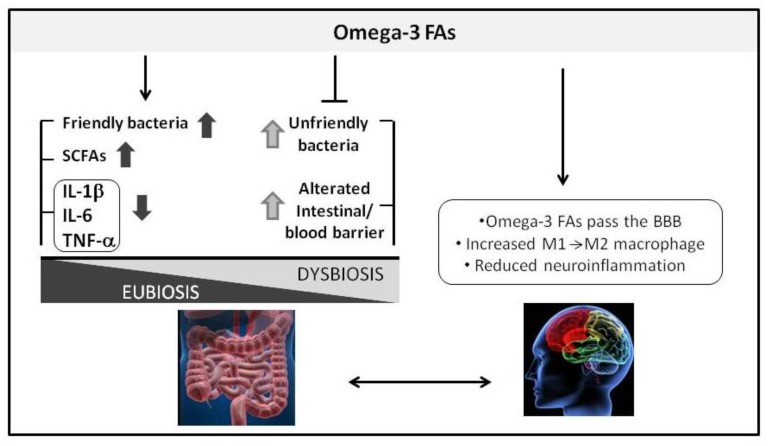
Possible effects of an omega-3 fatty acid (FA)-rich diet on microbiota and the brain. Omega-3 FAs lead to increases in host-friendly bacterial genera (*Eubacterium, Anaerostipes*, *Roseburia*, *Subdoligranulum, Coprococcus* and *Pseudobutyrivibrio*) associated with SCFA production.

Very recently, Watson et al., by next-generation sequencing (NGS) technology, described the effect of 4 g a day of mixed DHA/EPA supplement for eight weeks on the microbiota composition of 20 middle-aged healthy individuals [73]. Differences were not observed in the *Firmicutes*/*Bacteroidetes* phyla ratio, but differences were reported at the family and genus levels. This study highlighted the increased abundance of butyrate-producing bacterial genera (phylum *Firmicutes*, see Figure 2) after omega-3 FAs supplementation.

The Canola Oil Multicenter Intervention Trial was a double-blinded randomised crossover clinical study carried out on 25 volunteers who were enrolled based on the presence of at least one of the metabolic syndrome risk factors [74]. Five different unsaturated oil blends were used for 30 days, among these conventional canola oil and DHA-enriched high oleic canola oil (containing about 3.5 g DHA). Results, in line with previous work [73], showed no significant differences at the phylum level. However, after DHA dosing, a reversible increase in the abundance of so-called ‘beneficial’ bacterial genera, such as *Bifidobacterium*, *Lachnospira*, *Roseburia* and *Lactobacillus* (Figure 1), was observed during one or both types of omega-3 FAsA interventions. This is consistent with data obtained in mice.

In a case report, Noriega et al., using NGS, described the results of a diet treatment in a 45-year-old healthy man after two weeks of daily supplementation with 600 mg of omega-3 PUFA using fish protein: the *Firmicutes/Bacteroidetes* phyla ratio increased and *Actinobacteria* decreased. At the genera level, a reduction in Faecalibacterium versus an increase in *Blautia*, *Coprococcu*s, *Roseburia*, *Subdoligranulum* and *Ruminococcus* genera was observed. This change in microbiota composition led to greater SCFA production. After two washout weeks, the authors observed a reversed trend, confirming that the gut microbiota was susceptible to dietary change, as a dynamic ecosystem [75]. This result highlighted a temporal shift in the composition of microbial communities: an omega-3 FA dietary supplementation determined a marked and notable increase in genera associated with beneficial effects in the host (Figure 2) [75]. 

Recently, Menni et al. correlated DHA circulating levels with DHA dietary intake determined by a Food Frequency Questionnaire using a cohort of 876 middle-aged and elderly women. The DHA intake of 350 mg/day was found to positively correlate with SCFA-producing bacteria as demonstrated by using next-generation DNA sequencing (i.e., *Lachnospiraceae* family) [76]. 

The Pilchardus Study was a multicentre randomised clinical trial in patients diagnosed with type 2 diabetes who had not received any kind of treatment with antidiabetic drugs (using quantitative real-time polymerase chain reaction on target bacterial indicators). After a sardine-enriched diet in drug-naïve patients, the authors described a change in the gut microbiota at the phylum level, with a decrease in the *Firmicutes/Bacteroidetes* ratio and an increase in the Prevotella genus [77]. This evidence was also described by Costantini et al., 2017 [16].

In conclusion, omega-3 FAs supplementation increases SCFA production shifting gut microbiota balance towards a healthy status (eubiosis). The mechanism that underlies bacterial selection of omega-3 FAs has not yet been determined.

### 3.2. Omega-3 Fatty Acids: A Nutritional Strategy in Alzheimer’s Disease Treatment? 

As previously described, investigations into dietary impact on the gut microbiota show an interactive relationship between altered microbiota composition and the development of neurodegenerative diseases [46,78,79]. Given the effect of omega-3 FA in microbiota and given its anti-inflammatory effects, omega-3 FAs supplementation has emerged as a candidate for the prevention and management of AD.

Furthermore, meta-analyses studies described significantly lowered DHA levels in the blood as well as lower brain/cerebrospinal fluid (CSF) concentrations in patients with AD [77]. Several groups also reported that a reduction in cerebral omega-3 was linked to cognitive aging and to an increase in the progression of AD [80,81].

To analyse the impact of omega-3 FAs supplementation on AD cognitive impairment, we systematically collected articles found in MEDLINE. The search strategy combined the term “omega-3” AND “Alzheimer” and identified 394 papers. After screening, full-text examination, eligibility analysis we finally selected six papers, reported in Table 2. We selected only randomized control trials (RCT) and we included only studies where omega-3 FA supplementation combined DHA and EPA. Studies describing the effects of EPA and DHA used in combination with other substances were excluded (Table 2). All studies selected reported the effects on mild to moderate AD patients of an omega-3 FA supplement duration between 4 and 18 months.

Sample size varied from 17 to 402 women and men, 55–90 years old with mild-to-moderate AD, according to the Mini-Mental State Examination (MMSE) questionnaire (Table 3). The supplementation time varied from four months to 18 months. In all studies patients were subjected to the medication for AD prior to the beginning of intervention. In all studies, placebo groups received soy oil, olive oil, corn oil, or an isocaloric product. 

The most frequent questionnaires that were used in these studies to assess cognitive performances were Alzheimer’s Disease Assessment Scale (ADAS-cog) and MMSE (Table 3). 

Freund-Levi et al. [18] in a randomized, double-blind placebo-controlled clinical trial assessed omega-3 FAs supplementation in 174 patients (mean age: 74 ± 9 years) with mild-to moderate AD. The treated group received each day 1720 mg DHA and 600 mg EPA for six months, whereas the placebo group received each day 4 g of corn oil, containing 2.4 g of linoleic acid. ADAS-cog, MMSE and Clinical Dementia Rating Scale (CDR) did not show a statistically significant difference between the two groups. A significant difference was measured in the first six months only in a small number of patients with very mild AD. 

In a second paper published from the same research group using the same sample [82], neuropsychiatric behaviour was assessed by means of Neuropsychiatric inventory (NPI), Disability Assessment for Dementia Scale (DAD) and Montgomey Asberg Depression Rating Scale (MADRS). No significant differences were observed in the depression scale between the omega-3FAs group vs. the placebo group (NPI or MADRS score) and no statistically significant difference was seen in DAD score changes. 

Quinn et al. [83] assessed 402 patients with mild-to moderate AD (mean age: 76 ± 8.7 years). The intervention group received each day 2 g of DHA and the placebo group received soy or corn oil for 18 months. No significant effect was observed in cognitive and functional decline in ADAS-cog, CDR, Alzheimer Disease Cooperative Study—Activities of Daily Living (ADCS-ADL), NPI, and in MMSE on the 18-month follow-up. Some patients (*n* = 102) received volumetric magnetic resonance imaging (MRI) and there was no change in brain atrophy decline between the two groups. ADCS-ADL assess basic living skills such as eating and bathing. A variant of this method is Alzheimer Disease Cooperative Study—Instrumental Activities of Daily Living (ADCS-IADL) that measure more complex daily functions such as using the telephone, shopping or cooking (Table 3). Quinn et al. [83] used the full scale and did not find statistically significant differences between the placebo and the intervention group.

Shinto et al. [84] studied 34 subjects older than 55 years with probable AD in a pilot study organized in a three-arm, parallel group, double-blind placebo-controlled trial. One group received only omega-3 FAs (975 mg EPA and 675 mg DHA), a second group received the same supplement with the addition of alpha lipoic acid (600 mg ALA) while the placebo group only received soy oil. Supplementation lasted 12 months. As a result, there were no differences in ADAS-cog and ADCS-ADL between placebo and both supplemented groups. There was no difference between placebo and omega 3 FA group in MMSE scores, whereas a statistically significant difference was found between the placebo group and the omega-3 FAs + ALA group. Shinto et al. [84] used a modified version of the ADCS-ADL scores separating the two skills: basic living skills, by means of ADCS-ADL, and more complex daily functions, by means of ADCS-IADL. Whereas in ADCS-ADL there were no differences between the three examined groups, both groups that received omega-3 FAs evidenced a delay in functional decline when compared with placebo with the ADCS-IADL scores. Therefore, this scale that measures more complex tasks proved to be more sensitive to AD progression than ADCS-ADL.

Phillips et al. [85] assessed omega-3 FAs supplementation in 57 subjects with cognitive impairment and 19 with AD (mean age 71.1 ± 4.8 years). For four months, the treated group received each day 625 mg DHA and 600 mg EPA. The placebo group received olive oil. There was no statistical difference in MMSE between the two groups.

Eriksdotter et al. [86] reported on an investigative part of the Omega AD study, a randomized, double-blind placebo-controlled trial in 174 patients (mean age: 74 ± 9 years) with mild to moderate AD according to the Diagnostic and Statistical Manual of Mental Disorders IV edition criteria (DSM-IV). The authors demonstrated a dose-response relationship between omega-3 FA levels in plasma and the preservation of cognitive functions after the six-month administration of a DHA-rich omega-3 FAs supplementation. The preservation of cognitive performance, assessed by Alzheimer’s Disease Assessment Scale–Cognition (but not a Mini-Mental State Examination) in mild forms of AD or of mild cognitive impairment (MCI) was significantly associated with increasing plasma omega-3 FA levels over time: the higher the omega-3 FA plasma level rose, the lower was the rate of cognitive deterioration. 

We summarize as follows some of the critical issues which emerged from these studies:

1. In two cases the sample size was too small, limiting statistical power [84,85];
2. It is difficult to establish comparative analysis because of heterogeneity in composition, dosage and duration of supplementation;
3. It is difficult to establish comparative analysis because of heterogeneity of methods used to assess cognition (Table 3);
4. The placebo group in one study [85] included olive oil, a source of monounsaturated fatty acid. As previously demonstrated by other authors, monounsaturated fatty acids are inversely correlated with cognitive decline and therefore cannot be considered a totally inert substance on cognition performance;
5. In all presented studies, the subjects received medications for AD along with omega-3 FAs. This condition could also contribute to good outcomes in control group, hiding and/or slowing down the physiological decline in AD;
6. The duration of treatment with omega-3 FAs could be too short for a chronic disease such as AD;
7. Omega-3 FAs dosage could be insufficient to provide significant benefits.

In summary, the evidence reported herein indicates that the effect of omega-3 FAs on cognitive outcomes in AD was either insufficient or low-strength. Most studies do not find statistically significant results when the intervention group is compared to the placebo group. Nevertheless, according to MMSE scores assessments, omega-3 FAs supplementation seems to demonstrate efficacy in very mild AD [18]. MMSE measures cognitive performances like memory, orientation, language, attention, calculation, and visual construction. In particular, the analysis of subjects with mild AD demonstrated that the cognitive improvement concerns mainly memory functions. Memory deficiency is considered an AD hallmark symptom that most times represents the beginning of the clinically-relevant disease. These data are in agreement with findings from epidemiological observational studies. Several cohort studies suggest that fish consumption, being a source of omega-3 FAs, would have a role in AD prevention but not as adjuvant once the disease has already established [87,88,89,90]. 

Furthermore, it is possible that omega-3 FAs improve more complex activities of daily living. This data has to be confirmed in further trials.

Macrophages of patients with MCI and AD are defective in Aβ phagocytosis and show low resistance to Aβ-induced apoptosis. This functional defect of macrophages in AD is improved by omega-3 FAs. Very recently, Famenini et al. described how a drink containing omega-3, antioxidants and resveratrol maintained active Aβ_1-42_ phagocytosis, prevented brain amyloidosis, thus preserving cognition. As concerns the cellular mechanisms involved in the process, omega-3 FAs supplementation would modulate macrophages to the pro-phagocytic M1–M2 type and improve cognition in patients with MCI [17]. These macrophages exert an effective unfolded protein response against endoplasmic reticulum stress. After taking this supplement, several patients with MCI maintained a stable cognitive status for three years. However, the validity of this study was limited by its small size and the uncontrolled experimental design. Further randomised clinical trials are required to investigate this observational data [91].

Fiala et al. [91] discussed the use of immunotherapy for the treatment of MCI by omega-3 FAs supplementation. Based on a previous study, the authors described how omega-3 supplementation was successful in: (i) modulating a macrophage phenotype from an intermediate M2 phenotype (anti-inflammatory); (ii) attenuating Aβ_1-42_ secretion in AD; and (iii) reducing cytokine secretion in human neural cells with the consequent formation of a novel, DHA-derived 10,17S-docosatriene DHA (NPD1) that promoted brain cell survival by inducing gene-expression programs of anti-apoptotic and neuroprotective pathways, especially in the hippocampus, a structure involved in memory [92,93,94]. NPD1 also has beneficial immune effects under inflammatory and degenerative conditions [95]. Despite the powerful biochemical and immunological effects of omega-3 FAs supplementation demonstrated by several kinds of in vitro and in vivo activity, the quality of omega-3 products available on the market are poorly standardised and of low quality. Thus, the authors suggest that a combination drink called “Smartfish” containing an emulsion of omega-3 and other fatty acids, combined with the antioxidants, vitamin D3 and resveratrol maintained active Aβ1-42 phagocytosis, prevented brain amyloidosis leading to a preservation of cognition.

## 4. Probiotic Strategy in Alzheimer’s Disease Treatment? 

Some probiotics could enable the effective therapeutic management of neurodegenerative disorders.

Several studies have described the immunomodulatory action of some probiotic bacteria able to reduce the production of pro-inflammatory cytokines and to increase the activity of the M2 phenotype macrophages.

It has been recently demonstrated in vitro that a probiotic mixture containing *Lactobacillus rhamnosus*, *Bifidobacterium lactis* and *Bifidobacterium longum* (Serobioma, provided by Bromatech, s.r.l., Milan, Italy) exerted an anti-inflammatory and immunomodulatory action on THP-1 cells [96]. In particular, they reported a 70–80% reduction in the production of pro-inflammatory cytokines such as IL-1β and IL-6 and a statistically significant increase in the production of anti-inflammatory cytokine IL-10 by using the transwell model.

In animal models, some probiotics appear to influence the central nervous system (CNS) via the gut–brain axis [97].

Liu et al. reported that treatment with *Lactobacillus plantarum* PS128 normalized anxiety-like behaviour and reduced inflammatory cytokine levels in the plasma in stressed mice, giving psychotropic effects [98].

Moreover, treatment with VSL#3 (provided by Ferring Pharmaceutical Spa, Milan, Italy) that is a probiotic mixture containing eight different Gram-positive bacterial species improved neuronal functions and plasticity in young and aged rats. VSL#3 altered the expression of genes in the brain tissue such as BDNF and synapsin that are associated with inflammation and neural plasticity [99].

The effect of *Bifidobacterium breve* strain A1 on the behaviour and physiological processes of AD in mice models was investigated by Kobayashi et al. [100]. Authors found that the administration of *B. breve* A1 to AD mice increased spatial memory performances and suppressed the hippocampal expression of inflammation and immune-reactive genes that are induced by Aβ.

In a randomized, double-blind and controlled trial that enrolled 60 AD patients, it has been reported that a 12-week consumption of a mixture of probiotics improved cognitive function (measured by MMSE score) and metabolic status (measured by MDA, hs-CRP, insulin metabolism markers, serum levels of triglycerides and VLDLs) [101].

It is important to underline that, although the probiotic approach could be a strategy to improve gut microbiota composition, it did not show permanent effects able to treat dysbiosis.

## 5. Conclusions

Our gut microbiota plays a crucial role in the structural integrity of the intestinal mucosa through the production of SCFAs, such as butyrate, propionate and acetate. An altered microbiota may lead to a dysbiotic condition with the production of immunogenics endotoxins such as LPS and increasing intestinal permeability (the so-called leaky gut). This condition triggers an inflammatory response. The activation of microglia and peripheral monocytes that cross the BBB produces inflammatory cytokines and neurotoxic molecules that trigger neuroinflammation, as reported also in AD. Further studies are needed to characterize the gut microbiota composition in AD patients.

Several studies evidence how omega-3 FAs supplementation exerts a significant effect on microbiota composition, through a modulatory effect that increases butyrate producer bacteria. In this sense, we may consider omega-3 FAs as a prebiotic strategy for a healthy gut microbiota.

Despite this promising effect, there is little evidence regarding the beneficial effect of omega-3 FAs supplementation in AD’s cognitive decline. After a systematic revision of the literature, we selected six randomized control trials on mild to moderate AD patients where omega-3 FA supplement duration was between four and 18 months. Those short-term randomized trials with small sample size do not show any effect on cognitive improvement and therefore, longer term trials with longer omega-3 FA supplementation in early stage AD are warranted. Some studies reported that only mild cognitive decline in MCI could be affected by omega-3 FA supplementation. In this review, we report some of the critical issues which emerged from the studies.

Furthermore, we also believe it will be necessary to improve the quality and standardization of omega-3 FAs supplements available on the market, to study dose-dependent and time-dependent effects of nutritional supplements. It will also be necessary to quantify serum omega-3 FAs before and after supplementation in AD patients.

In conclusion, randomized controlled trials considering the critical issues underlined in this review are warranted.

## Figures and Tables

**Table 1:** Studies investigating the omega-3 fatty acid influence on human gut microbiota.

Author	Studied Population	Study Design	Supplement	Supplementation Duration	Methods	Results
Watson et at., 2017, [73]	20 middle-aged healthy people	RCT	4 g of mixed DHA/EPA as capsules and drink	8 weeks	NGS of 16S rRNA gene, V4 region	At phylum level: no difference for Firmicutes/Bacteroidetes ratio. At family level: increase in Clostridiaceae, Sutterellaceae and Akkermansiaceae. At genus level: increased abundance of Bifidobacterium Oscillospira and reduction of Caprococcus and Faecalibacterium genera. In drink group increased abundance of Lachnospira and Roseburia genera.
Pu et al., 2016, [74]	25 people with risk of metabolic syndrome	RCT	60 g of different unsaturated oil blend	30 days	Pyrosequncing of 16S rRNA gene, V1-V3 regions	At phylum level: no difference. Reversible increase in Bifidobacterium, Lachnospira, Roseburia, and Lactobacillus
Noriega et al., 2016, [75]	Case report	one healthy 45-year-old man	600 mg PUFA by fish protein diet	2 weeks	NGS of 16S rRNA gene, V4 region	At phylum level: increase in Firmicutes and decrease in Bacteroidetes and Actinobacteria. At genus level: increase in Blautia, Roseburia, Coprococcus, Ruminococcus, and Subdoligranulum. Decrease in Faecalibacterium
Menni et al., 2017, [76]		876 middle-aged and elderly women	350 mg/day of DHA		NGS of 16S rRNA gene, V4 region	At phylum level: increase in Lachnospiraceae.
Ballego et al., 2016, [77]		32 patients with type 2 diabetes	100 g of sardines (about 3 g di DHA + EPA/day)	5 day a week for 6 months	qPCR	At phylum level: Firmicutes/Bacteroidetes ratio decrease. At genus level: Prevotella increase

**Table 2:** Selected clinical trials assessing the impact of omega-3 FAs supplementation in AD patients.

Author	Number of Patients (Dropout Excluded)	Age of Patients	Type of Patients	Study Design	Supplement	Supplementation Duration (months)	Methods	Conclusions
Freund-Levi et al., 2006, [18]	174	74 ± 9	Mild to moderate AD	RCT	1.7 g DHA + 0.6 g EPA	6–12	MMSE, ADAS-cog CDR	Did not delay the cognitive decline except in a subgroup with very mild AD
Freund-Levi et al., 2008, [82]	174	74 ± 9	Mild to moderate AD	RCT	1.7 g DHA + 0.6 g EPA	6–12	NPI, MADRS, DAD, CGP	Does not ameliorate neuropsychiatric symptoms
Quinn et al., 2010, [83]	402	76 ± 8.7	Mild to moderate AD	RCT	2 g DHA	18	MMSE ADAS-cog CDR ADCS-ADL NPI MRI	Does not slow the cognitive and functional decline in mild to moderate AD
Shinto et al., 2014, [84]	34	>55	probable AD	RCT	675 mg DHA + 975 mg EPA	12	MMSE ADAS-cog ADCS-ADL	Decrease the rate of decline in MMSE
Phillips et al., 2015, [85]	76	71 ± 4.8	57 with cognitive impairment and 19 with AD	RCT	600 mg DHA + 625 mg EPA	4	MMSE	Negligible benefits on mood and cognition in AD
Eriksdotter et al., 2015, [86]	174	74 ± 9	Mild to moderate AD	RCT	1.7 g DHA + 0.6 g EPA	6	MMSE ADAS-cog	Stabilizes the cognitive performance of AD subjects.

**Table 3:** Methods used for cognitive assessment in AD patients.

Methods	Description
**MMSE** Mini-Mental State Examination	Evaluates memory, orientation, language, calculation, attention and visual construction. It is used in clinical practice. Scores range between 0 and 30. Typically, an A cut-off score of 23 or 24 has been used to define significant cognitive impairment.
**ADAS-cog** Alzheimer’s Disease Assessment Scale—Cognitive section	It is a reliable and sensitive psychometric method for the assessment of cognitive function in dementia. It is used to evaluate changes over time. It consists of 11 items and a score scale between 0 (no impairment) and 70 (very severe impairment).
**ADCS-ADL** Alzheimer Disease Cooperative Study—Activities of Daily Living	It measures the functional ability to assess basic living skills of daily life such as bathing, eating.
**ADCS-IADL** Alzheimer Disease Cooperative Study—Instrumental Activities of Daily Living	It measures the functional ability to assess complex skills of daily life such as preparing a meal, using the telephone, shopping.
**CDR** Clinical Dementia Rating Scale	It is based on caregiver interview. It assesses memory, judgment, orientation. Dementia is classified into questionable, mild, moderate, and severe.
**NPI** Neuropsychiatric Inventory	It assesses dementia-related behavioural symptoms.
